# Ginkgo Biloba Extract Is Comparable With Donepezil in Improving Functional Recovery in Alzheimer’s Disease: Results From a Multilevel Characterized Study Based on Clinical Features and Resting-State Functional Magnetic Resonance Imaging

**DOI:** 10.3389/fphar.2021.721216

**Published:** 2021-08-03

**Authors:** Yu Zheng, Yi Xie, Ming Qi, Ling Zhang, Wei Wang, Wanrong Zhang, Liju Sha, Jiawen Wu, Wanting Li, Ting Wu

**Affiliations:** ^1^ Department of Rehabilitation Medicine, The First Affiliated Hospital of Nanjing Medical University, Nanjing, China; ^2^ Division of Brain Rehabilitation, Department of Neurology, The First Affiliated Hospital of Nanjing Medical University, Nanjing, China; ^3^ Departments of Radiology, The First Affiliated Hospital of Nanjing Medical University, Nanjing, China; ^4^ Department of Neurology, The First Affiliated Hospital of Nanjing Medical University, Nanjing, China

**Keywords:** ginkgo biloba extract, donepezil, Alzheimer’s disease, resting-state functional magnetic resonance imaging, functional recovery

## Abstract

**Background:** Ginkgo biloba extract (GBE) and donepezil have been reported to be effective in patients with Alzheimer’s disease (AD). Nonetheless, how these drugs impact spontaneous brain activities and how they consequently improve functional recovery are currently unclear.

**Objectives:** This study was to explore the efficacy of GBE vs. donepezil and their add-on efficacy on functional recovery and the adaption of spontaneous brain activities following pharmacologic treatment in patients with AD.

**Methods:** Patients with AD were enrolled and assigned to the GBE group (*n* = 50), the donepezil group (*n* = 50), or the combined group (*n* = 50). Neuropsychological assessments, including minimum mental state examination (MMSE), Alzheimer’s disease assessment scale-cognition (ADAS-Cog), instrumental activity of daily living (IADL), geriatric depression scale (GDS), neuropsychiatric inventory (NPI), and quality of life in Alzheimer’s disease (QOL-AD), were conducted at baseline, 1 month, 3 months, and 6 months. Resting-state functional magnetic resonance imaging (rs-fMRI) was collected to compare the amplitude of low-frequency fluctuation (ALFF), percent amplitude of fluctuation (PerAF), regional homogeneity (ReHo), and degree centrality (DC) at baseline and 6 months.

**Results:** No major significant differences were detected in all comparisons between groups across all follow-up time points. For intragroup comparison, MMSE and ADAS-Cog scores differed significantly across all follow-ups in three groups. The combined group showed significant improvement of GDS scores between baseline and 6 months (*p* = 0.007). The GBE group (*p* = 0.044) and donepezil group (*p* = 0.012) demonstrated significant improvement of NPI scores between baseline and 6 months. Significant correlations were observed between IADL and ALFF in the right gyrus rectus (*p* = 0.03) and in the left superior cerebellum gyrus (*p* = 0.01), between GDS and ALFF in the right middle temporal gyrus (*p* = 0.01), between NPI and PerAF in the left fusiform gyrus (*p* = 0.03), and between MMSE and ReHo in right superior frontal gyrus (*p* = 0.04).

**Conclusion:** GBE was comparable with donepezil in the improvement of functional recovery in patients with AD while the combined application of GBE and donepezil seems unnecessary. GBE-mediated improvement of functional recovery was characterized by decreased ALFF values in the right gyrus rectus and decreased PerAF values in the left fusiform gyrus. These featured variations of imaging metrics in specific brain regions may serve as biomarkers in the monitoring of the therapeutic efficacy of GBE.

## Introduction

As one of the most prevalent causes of dementia, Alzheimer’s disease (AD) is an irreversible neurodegenerative disorder and is characterized by progressive cognitive and intellectual deficits (Reitz et al., 2011). Currently, approximately 47 million people suffer from AD worldwide, and it is expected to increase to more than 130 million by 2050 (Alzheimer's Disease International, 2015). The residual effect of AD may have a devastating personal and financial impact on individuals, families, and society.

Due to the difficulties in participating in physical and psychological interventions, patients with moderate-to-severe AD are more likely to be treated with pharmacologic strategies. Unfortunately, only several pharmacological agents, either preclinical or licensed, are currently available for the treatment of AD (Arvanitakis et al., 2019). The acetylcholinesterase inhibitors (e.g., donepezil) can specifically inhibit the acetylcholinesterase enzyme in the central nervous system, thereby promoting increases in acetylcholine abundance at the synaptic cleft for cholinergic neurotransmission. Based on a recently published Cochrane review, donepezil was proved to be a promising agent that has benefits on cognitive function, activities of daily living, and global impression scales (Birks and Harvey, 2018). Differentiated from acetylcholinesterase inhibitors, the Ginkgo biloba extract (GBE) is hypothesized to act on amyloid *β*-induced hippocampal neuron dysfunction and death, amyloid *β* aggregation, and neurogenesis (Bastianetto et al., 2000; Luo et al., 2002; Tchantchou et al., 2007). Recently, GBE has been extensively tested for treating cognitive impairment in patients with AD, while only limited clinical efficacy was demonstrated (Canter and Ernst, 2007; DeKosky et al., 2008; Vellas et al., 2012). Based on this condition, well-designed and executed clinical trials are warranted. Its efficacy needs to be further clarified when comparing its effects with conventional pharmacologic therapy. Furthermore, the question that, whether GBE adds benefit for patients already taking conventional drugs, is hopefully to be answered.

Afterward, the upcoming question is that how these agents interact with the brain. Specifically, it remains unknown whether the local spontaneous brain activities after pharmacologic treatment capture the neural recovery underlying global functional recovery as assessed by standardized measures used in AD clinical practice. The answers may further assist clinicians to understand which agents are effective overall and the relative efficacy of different agents. Therefore, there is an urgent need for sensitive biomarkers to detect a signal of pharmacologic efficacy. A number of major reviews on brain region disruption as assessed with functional magnetic resonance imaging (fMRI) have shown distinct patterns of brain region disruption across the major neurodegenerative diseases (Bozzali et al., 2011; Di Perri et al., 2016; Guo et al., 2016; Yang et al., 2020). It has been achieved consensus that the progression of AD induced symptoms follows a relatively stereotyped order: episodic memory loss occurs first, followed by semantic memory loss, aphasic, apraxic, and visuospatial symptoms, and finally motor and visual deficits (Dubois et al., 2007). The role of fMRI in this aspect is to link the functional impairment to the specific brain region. In a similar way, it can also demonstrate the functional adaption following pharmacologic treatment based on the featured fluctuations of blood oxygen level dependent (BOLD) signaling. With this advanced neurophysiological technique, the impact of pharmacologic treatment on spontaneous brain activities can be investigated noninvasively and then spread to functional recovery.

Based on the above perspectives, the aim of the current trial is 1) to compare the efficacy of GBE vs. donepezil on cognition, behavioral function, psychological function, and quality of life (QoL); 2) to explore the add-on efficacy of GBE with donepezil; and 3) to provide an overview of findings on adaption of spontaneous brain activities following pharmacologic treatment in patients with AD.

## Materials and Methods

### Study Design and Participants

This cohort study was a secondary analysis of data collected by an ongoing pragmatic, controlled, three-arm, parallel group, randomized controlled clinical trial, which was prospectively registered at the Clinical Trial Registry (https://clinicaltrials.gov): NCT03090516, August 5, 2019. The trial protocol was developed according to the Consolidated Standards of Reporting Trials (CONSORT) statements for pragmatic trials and has been reviewed and approved by the Research Ethics Committee at the First Affiliated Hospital of Nanjing Medical University (Reference number: 2016-SR-134). In accordance with the Declaration of Helsinki of 1964 as revised in 2013, the International Conference of Harmonization Guidelines for Good Clinical Practice and the requirement of the local ethics committees, written informed consent was obtained from all enrolled participants.

The target population for this study were those who met the consolidated inclusion criteria: 1) aged 50–85 years and right-handed; 2) diagnosed with AD or MCI according to the NINCDS/ADRDA guidelines (Dubois et al., 2007); 3) CT or MRI performed within 1 year potentially indicating AD or MCI (Planche et al., 2020); 4) MMSE score of 27 or less (Cummings, 1993); 5) able to follow medical instruction or assessment requirement; and 6) signed informed consent. The exclusion criteria were as follows: 1) diagnosed with vascular dementia according to the NINDS-AIREN criteria (Roman et al., 1993); 2) modified Hachinski ischemic score of 4 or more (Rosen et al., 1980); and 3) with major depression, schizophrenia, cerebrovascular diseases, Parkinson’s disease, or other systemic and neurodegenerative diseases.

One hundred and fifty eligible patients were enrolled and assigned into: 1) the Ginkgo biloba extract (GBE) group (*n* = 50), orally received 150 mg GBE three times daily for 6 months; 2) the donepezil group (*n* = 50), orally received 5 mg donepezil once daily for 6 months; and 3) the combined group (*n* = 50), orally received both GBE (150 mg three times daily) and donepezil (5 mg once daily) for 6 months.

### Assessments

Baseline demographics and clinical characteristics, collected directly from the patients or the medical documents, are as follows: gender, age, education length, disease subtype, AD/MCI history, family history of AD/MCI; comorbidity, ApoE genotype, modified Hachinski score (MHIS), Hamilton anxiety scale (HAMA) score, and clinical dementia rating (CDR) score (Thompson, 2015; Woolf et al., 2016).

Apart from the above variables, data in terms of minimum mental state examination (MMSE), Alzheimer’s disease assessment scale-cognition (ADAS-Cog), instrumental activity of daily living (IADL), geriatric depression scale (GDS), neuropsychiatric inventory (NPI), and quality of life in Alzheimer’s disease (QOL-AD) were collected at baseline, 1 month, 3 months, and 6 months. In addition, images and data of resting-state functional magnetic resonance imaging (rs-fMRI) were collected at baseline and 6 months. Detailed information of the above assessments is provided as follows.

### Minimum Mental State Examination

MMSE contains items assessing a wide range of cognitive functions, including orientation to time and place, concentration, language functions (following a three-step command, repeating a difficult phrase, naming high-frequency items, following a written command), construction, verbal learning, and short-delay recall. MMSE ranges from 0 to 30 with a higher score indicating better cognitive function. The cutoffs for AD and MCI are 24 and 27, respectively (Malloy et al., 1997).

### Alzheimer’s Disease Assessment Scale-Cognition

ADAS-Cog is consisted of the following 11 items: orientation (0–8), word recall (0–10), word recognition (0–12), commands (0–5), naming objects and fingers (0–5), ideational praxis (0–5), constructional praxis (0–5), ability of remembering (0–5), expressing (0–5), comprehension (0–5), and word finding (0–5). It is scored 0–70, and a higher score indicates poor performance (Doraiswamy et al., 2001).

### Instrumental Activity of Daily Living

IADL contains 14 items of instrumental activity of daily living: laundry, shopping, bathing, brushing hair and teeth, light housework, meals, walking, managing money, managing medications, dressing, transferring, using the phone, toileting, and eating. They are rated as follows: 1: can do, 2: some difficulty but can do, 3: need some help, and 4: cannot do on their own. A higher single score indicates poor ability of daily living, and a total score of higher than 16 indicates different degrees of functional decline (LaPlante, 2010).

### Geriatric Depression Scale

Thirty questions are included in the GDS and answered with yes or no. Positive answers in 20 out of 30 questions indicate presence of depression (e.g., Have you given up many of your activities and interests?), while other 10 questions with negative answers indicate presence of depression (e.g., Are you generally satisfied with your life?). The cumulative score is rated and classified with 0–9 as normal, 10–19 as mildly depressed, and 20–30 as severely depressed (Defrancesco et al., 2018).

### Neuropsychiatric Inventory

NPI is used to assess 12 symptoms reflecting behavioral function including delusions, hallucinations, agitation/aggression, dysphoria, anxiety, euphoria, apathy, disinhibition, irritability/lability, and aberrant motor activity. The absence of symptom is scored as 0. Both the frequency (1: occasionally, 2: often, 3: frequently, and 4: very frequently) and the severity of each symptom (1: mild, 2: moderate, and 3: severe) are rated. A total NPI score is calculated with the frequency*severity as a multiplied score (0–144). A higher score indicates severer psychobehavioral dysfunction and the cut off of 24 or more indicates a clinically significant psychobehavioral dysfunction (Vik-Mo et al., 2020).

### Quality of Life in Alzheimer’s Disease

The score of QOL-AD is computed by adding the following 13 items with each item scored 1–4: physical health, energy, mood, living situation, memory, family, marriage, friends, self as a whole, ability to do chores around the house, ability to do things for fun, money, and life as a whole. It is scored 13–52, and a higher score indicates higher QOL (Logsdon et al., 2002).

### Resting-State Functional Magnetic Resonance Imaging

#### Data Acquisition

rs-fMRI data were collected at baseline and 6 months. Scanning was performed on a Siemens Magnetom Trio 3.0T MRI System (Siemens AG, Erlangen, Germany) using a standard birdcage head transmit and receive coil. Functional images were acquired using a single-shot, gradient-recalled echo planar imaging sequence [repetition time (TR) = 2,000 ms; echo time (TE) = 30 ms; flip angle (FA) = 90°]. A total of 33 transverse slices [field of view (FOV) = 256 × 256 mm^2^; in-plane matrix = 64 × 64; slice thickness = 4 mm; inter-slice gap = 1 mm; voxel size = 4 × 4 × 4 mm^3^] aligned along the anterior-posterior commissure line. For each patient, a total of 240 volumes were acquired, resulting in a total scan time of 480 s. Patients were instructed to simply rest with their eyes closed. The high-resolution 3D T1-weighted anatomical images were collected in a sagittal orientation using a magnetization-prepared rapid gradient-echo sequence (TR = 1,900 ms; TE = 2.52 ms; FA = 9°; FOV = 256 × 256 mm^2^; matrix size = 256 × 256; slice thickness = 1 mm; inter-slice gap = 0.5 mm; voxel size = 1 × 1 × 1 mm^3^; 176 slices).

#### Processing

Data processing was based on MATLAB R2014a platform, using DPABI software and SPM12 software to process the scanning data (http://www.fil.ion.ucl.ac.uk/spm/software/spm12) (Chao-Gan and Yu-Feng, 2010). The data analysis toolkit converted the original image in the DICOM format to NIFTI format, and then performed image preprocessing. The detailed preprocessing steps are as follows. The first 10 time points were removed to avoid nonequilibrium effects of magnetization allowing patients to adjust to the scanner noise. Slice timing and correction of head motion were then performed. Twenty-one patients were excluded due to their head movement exceeded 3° rotation or 3 mm translocation in any direction during scanning. The individual structural images were then coregistered to the mean functional image after motion correction by using a linear transformation. The transformed structural images were segmented into gray matter, white matter, and cerebrospinal fluid by using a unified segmentation algorithm. The motion-corrected functional volumes were spatially normalized to Montreal Neurologic Institute space and resampled to 3 mm*3 mm*3 mm voxels by using normalization parameters estimated during unified segmentation. Linear detrending processing was conducted to remove the linear signal drift. The individual-level regression analysis was conducted to minimize the influence of head motion (Friston-24 model) (Friston et al., 1996), whiter matter signal noise, and cerebrospinal fluid signal noise. A band-pass filter (0.01–0.08 Hz) was applied in percent amplitude of fluctuation (PerAF), regional homogeneity (ReHo) and degree centrality (DC) calculation but not in amplitude of low-frequency fluctuation (ALFF).

#### Calculation of Amplitude of Low-Frequency Fluctuation, Percent Amplitude of Fluctuation, Regional Homogeneity and Degree Centrality

ALFF was estimated based on Fast Fourier transform (FFT) using DPABI v4.0 (Chao-Gan and Yu-Feng, 2010). Each time course was then converted to frequency domain without band-pass filtering. Then, the square root of the power spectrum at each frequency was averaged across the filtered band (0.01–0.08 Hz). The ALFF of each voxel was then divided by the global mean of ALFF values (mALFF) for standardization.

PerAF of each voxel was estimated with the following equations:

$$PerAF=\frac{1}{n}\sum_{i=1}^{n}\left|\frac{X_{i}-\mu }{\mu }\right|\times 100\text{\%,}$$

$$\mu =\frac{1}{n}\sum_{i=1}^{n}X_{i}.$$

Here, “*X*” represents the signal intensity of the time point, “*n*” refers to the total number of time points of time course, and “*µ*” is the mean value of the time course.

With DPABI v4.0, the Kendall’s coefficient of concordance (KCC) of time course of every 27 nearest neighboring voxels was calculated to account for ReHo. To reduce the influence of individual variations, ReHo map normalizations were performed by dividing KCC across each voxel with the averaged KCC of the whole brain.

DC represents the sum of weights that shows node strength with a given voxel in weighted graphs. For each voxel, the BOLD time course was extracted, and the Pearson correlation coefficients with every other voxel in the brain were calculated. A matrix of Pearson correlation coefficients between a given voxel and all other voxels was generated to show the whole-brain functional connectivity matrix for each voxel. An undirected adjacency matrix was then generated by setting a threshold to each correlation at an *r* value more than 0.25 (Buckner et al., 2009; Wang et al., 2018; Wang et al., 2020). DC is defined as the sum of weights (r-values) of significant functional connections (*r* > 0.25) for each voxel. The DC value of each voxel was divided by the global mean of the DC values for standardization.

The standardized ALFF, PerAF and ReHo maps, and DC matrices were smoothed with Gaussian kernel (full width at half maximum FWHM = 6 mm).

### Statistical Analysis

Analyses for clinical data were performed using SPSS 20.0 (IBM Corporation, United States). Descriptive statistics (mean, standard deviation, and proportion) were utilized to demonstrate the distribution of the results with respect to statistical quantitative features. After testing the normality, the demographic data of the two experimental groups were compared with independent samples *t*-test and chi-square test. For the intergroup comparison, the continuous data, including MMSE, ADAS-Cog, IADL, GDS, NPI, and QOL-AD, were compared with repeated one-way ANOVA. For the intragroup comparison, repeated one-way ANOVA was applied for the comparison of differences between individual time points (baseline vs. 1 month, baseline vs. 3 months and baseline vs. 6 months). Post-hoc tests were conducted with the Bonferroni method. The statistical significance was determined with adjusted *p* value less than 0.05.

To examine differences of ALFF, PerAF, ReHo, and DC between baseline and 6 months, paired *t*-test was conducted using DPABI v4.0. To reduce the impact of confounding variables in the analysis, we performed paired *t*-tests with the mean framewise displacement as covariates (Jenkinson et al., 2002). Multiple comparison correction was performed based on the Gaussian random field theory (GRF, voxel-wise *p* < 0.005, cluster-wise *p* < 0.05, and two-tailed). For any measure (ALFF, PerAF, ReHo, or DC) showing post-intervention alterations, the Pearson correlation was used to predict associations between the value changes of the sphere, the peak coordinate of the significant discriminative cluster with a radius of 6 mm, with clinical neuropsychological changes. The correlations were considered significant at a threshold of *p* < 0.05.

## Results

### Sample Characteristics

**FIGURE 1:**
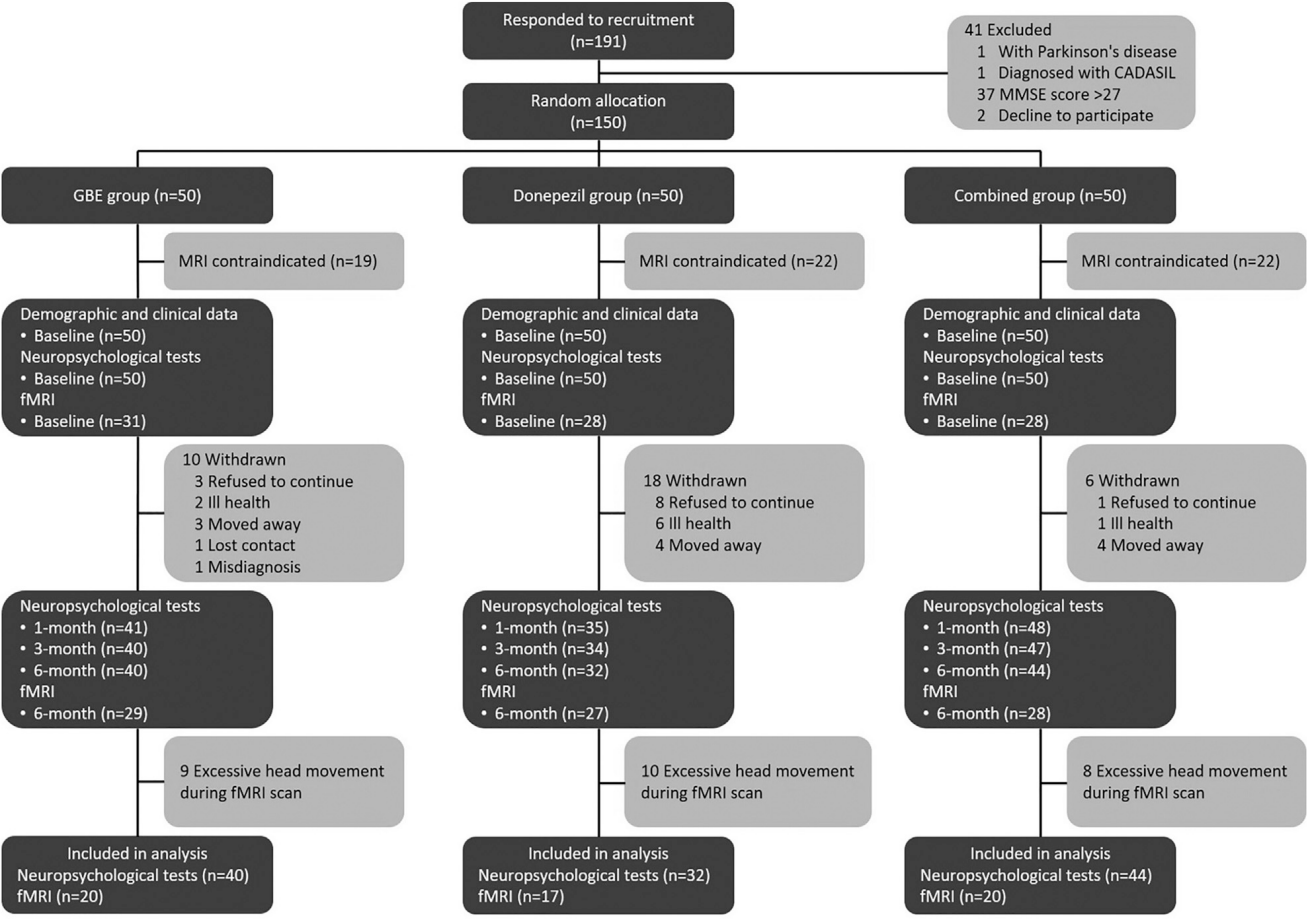
Study logistics of recruitment, assignment, intervention, and assessment. CADASIL: cerebral autosomal dominant with subcortical infarcts and leukoencephalopathy; MMSE: minimum mental state examination; GBE: Ginkgo biloba extract; MRI: magnetic resonance imaging; fMRI: functional magnetic resonance imaging.

A total of 191 patients were enrolled while 41 of them were excluded according to the results of eligibility assessment. Therefore, a total of 150 eligible patients participated the current study and received several follow-up assessments. Demographic and clinical characteristics of the sample were presented in Table 1. No significant differences were detected in all variables across three groups. Based on the results of quality check, 20 cases from the GBE group, 17 cases from the donepezil group, and 20 cases from the combined group provided sufficiently qualified fMRI data for further multi-model analysis. The study logistics of recruitment, assignment, intervention, and assessment were demonstrated in Figure 1.

**TABLE 1:** Clinical characteristics of the sample.

	GBE group (*n* = 50)	Donepezil group (*n* = 50)	Combined group (*n* = 50)	*p* value
Male, %	19 (38)	21 (42)	18 (36)	0.821
Age, mean (SD)	71.06 (7.65)	71.34 (8.23)	70.98 (6.71)	0.969
Education length in year, mean (SD)	12.92 (2.59)	12.39 (3.00)	12.58 (3.05)	0.650
Disease stage (MCI/AD), %	24/26	16/34	17/33	0.199
Disease duration in year, mean (SD)	2.39 (1.71)	2.31 (1.68)	2.57 (1.66)	0.746
Family history of AD, %	11 (22)	10 (20)	14 (28)	0.616
Complications, %				
Stroke or TIA history	8 (16)	5 (10)	6 (12)	0.656
Hypertension	9 (18)	17 (34)	9 (18)	0.092
Diabetes	3 (6)	8 (16)	3 (6)	0.140
Coronary atherosclerosis	1 (2)	2 (4)	2 (4)	0.813
Hypothyroidism	0 (0)	1 (2)	1 (2)	0.602
Asthma	0 (0)	2 (4)	0 (0)	0.132
APOE *ε*4+, %	6 (25)	3 (19)	5 (21)	0.898
MHIS, mean (SD)	0.96 (0.81)	0.78 (0.71)	0.86 (0.83)	0.518
HAMA, mean (SD)	2.48 (2.60)	1.72 (2.12)	2.00 (1.99)	0.237
CDR, mean (SD)	1.08 (0.27)	1.24 (0.43)	1.22 (0.42)	0.077

GBE: Ginkgo biloba extract; SD: standard deviation; MHIS: modified Hachinski score; MCI: mild cognitive impaired; AD: Alzheimer's disease; TIA: transient ischemic attacks; HAMA: Hamilton anxiety scale; CDR: clinical dementia rating.

### Changes of Neuropsychological Function Following Pharmacologic Treatment

Table 2 shows the intergroup comparison across baseline, 1 month, 3 months, and 6 months. Unfortunately, no significant differences were detected in all comparisons between groups across all follow-up time points except MMSE between the GBE group and donepezil group at 1 month (*p* = 0.019). For the intragroup comparison, MMSE and ADAS-Cog scores differed significantly in all three groups. Compared to the status at baseline, a gradual improvement of cognitive function was observed as time went by (Tables 3–5). IADL and QOL-AD scores changed marginally across four visits in all three groups although occasional significant differences were presented between baseline and 3-month evaluation of IADL in the GBE group and combined group (Tables 3, 5), and between baseline and 3-month evaluation of QOL-AD in the combined group (Table 5). Regarding the comparison of GDS scores between baseline and 6 months, only the combined group showed a significant difference (*p* = 0.007, Table 5). In addition, the GBE group (*p* = 0.044) and donepezil group (*p* = 0.012) demonstrated significant improvement between baseline and 6-month evaluation in terms of NPI scores (Tables 3, 4).

**TABLE 2:** Intergroup outcome comparison across four visits.

	GBE group (I)		Donepezil group (II)		Combined group (III)		*p* value*†*		
	Mean	SD	Mean	SD	Mean	SD	I vs. II	I vs. III	II vs. III
Baseline									
MMSE	23.72	2.86	22.32	3.40	22.10	4.35	0.159	0.077	1.000
ADAS-Cog	17.65	8.00	21.05	8.57	21.99	10.90	0.213	0.066	1.000
IADL	15.16	2.68	15.44	1.77	16.08	3.58	1.000	0.300	0.754
GDS	5.43	5.64	4.42	4.76	5.58	5.49	1.000	1.000	0.829
NPI	2.34	5.77	1.44	2.93	1.82	8.32	1.000	1.000	1.000
QOL-AD	31.06	6.85	32.90	6.05	33.14	6.85	0.502	0.357	1.000
1 month									
MMSE	26.20	2.65	23.54	4.45	24.16	4.87	0.019*	0.066	1.000
ADAS-Cog	14.41	6.13	19.19	9.44	18.13	11.04	0.078	0.177	1.000
IADL	14.83	1.88	15.63	1.96	16.69	3.09	0.475	0.293	1.000
GDS	3.10	3.13	3.69	3.50	3.86	3.81	1.000	0.927	1.000
NPI	0.78	2.23	0.37	1.03	0.82	2.73	1.000	1.000	1.000
QOL-AD	32.32	6.49	31.60	5.25	33.63	5.48	1.000	0.851	0.342
3 months									
MMSE	26.43	2.68	24.00	4.75	24.31	5.24	0.060	0.082	1.000
ADAS-Cog	12.73	6.05	17.07	9.73	17.16	11.37	0.153	0.091	1.000
IADL	14.63	1.56	15.32	1.98	15.65	3.21	0.668	0.160	1.000
GDS	3.40	4.58	4.29	5.17	3.33	3.60	1.000	1.000	0.997
NPI	0.83	2.25	0.47	1.85	1.16	3.24	1.000	1.000	0.703
QOL-AD	32.43	6.49	32.50	6.12	34.60	5.82	1.000	0.298	0.384
6 months									
MMSE	26.71	2.46	23.85	4.86	24.70	5.25	0.972	0.943	0.962
ADAS-Cog	12.31	5.43	16.36	9.09	16.54	10.76	0.119	0.081	1.000
IADL	14.64	1.82	15.26	2.22	15.50	2.89	0.737	0.286	1.000
GDS	4.20	5.32	4.67	5.65	3.43	5.00	1.000	1.000	0.795
NPI	0.52	1.52	0.21	0.77	0.52	1.56	0.870	1.000	0.859
QOL-AD	32.38	6.62	33.51	6.50	33.91	6.29	1.000	0.827	1.000

GBE: Ginkgo biloba extract; SD: standard deviation; MMSE: minimum mental state examination; ADAS-Cog: Alzheimer's disease assessment scale-cognition; IADL: instrumental activity of daily living; GDS: geriatric depression scale; NPI: neuropsychiatric inventory; QOL-AD: quality of life in Alzheimer's disease. †: Bonferroni pairwise comparison. *p < 0.05.

**TABLE 3:** Intragroup outcome comparison in the GBE group.

	Baseline		1 month		3 months		6 months		*p* value		
	Mean	SD	Mean	SD	Mean	SD	Mean	SD	1 m vs. BL	3 m vs. BL	6 m vs. BL
MMSE	23.72	2.86	26.20	2.65	26.43	2.68	26.71	2.46	<0.001*	<0.001*	<0.001*
ADAS-Cog	17.65	8.00	14.41	6.13	12.73	6.05	12.31	5.43	<0.001*	<0.001*	<0.001*
IADL	15.16	2.68	14.83	1.88	14.63	1.56	14.64	1.82	0.181	0.017*	0.194
GDS	5.43	5.64	3.10	3.13	3.40	4.58	4.20	5.32	0.001*	0.007*	0.184
NPI	2.34	5.77	0.78	2.23	0.83	2.25	0.52	1.52	0.056	0.057	0.044*
QOL-AD	31.06	6.85	32.32	6.49	32.43	6.49	32.38	6.62	0.197	0.105	0.138

GBE: Ginkgo biloba extract; SD: standard deviation; BL: baseline; 1 m: 1 month; 3 m: 3 months; 6 m: 6 months; MMSE: minimum mental state examination; ADAS-Cog: Alzheimer's disease assessment scale-cognition; IADL: instrumental activity of daily living; GDS: geriatric depression scale; NPI: neuropsychiatric inventory; QOL-AD: quality of life in Alzheimer's disease. *p < 0.05.

**TABLE 4:** Intragroup comparison of clinical scales in the donepezil group.

	Baseline		1 month		3 months		6 months		*p* value		
	Mean	SD	Mean	SD	Mean	SD	Mean	SD	1 m vs. BL	3 m vs. BL	6 m vs. BL
MMSE	22.32	3.40	23.54	4.45	24.00	4.75	23.85	4.86	0.007*	<0.001*	0.002*
ADAS-Cog	21.05	8.57	19.19	9.44	17.07	9.73	16.36	9.09	0.001*	<0.001*	<0.001*
IADL	15.44	1.77	15.63	1.96	15.32	1.98	15.26	2.22	0.360	0.099	0.539
GDS	4.42	4.76	3.69	3.50	4.29	5.17	4.67	5.65	0.048*	0.611	0.795
NPI	1.44	2.93	0.37	1.03	0.47	1.85	0.21	0.77	0.041	0.141	0.012*
QOL-AD	32.90	6.05	31.60	5.25	32.50	6.12	33.51	6.50	0.720	0.532	0.413

SD: standard deviation; BL: baseline; 1 m: 1 month; 3 m: 3 months; 6 m: 6 months; MMSE: minimum mental state examination; ADAS-Cog: Alzheimer's disease assessment scale-cognition; IADL: instrumental activity of daily living; GDS: geriatric depression scale; NPI: neuropsychiatric inventory; QOL-AD: quality of life in Alzheimer's disease. *p < 0.05.

**TABLE 5:** Intragroup comparison of clinical scales in the combined group.

	Baseline		1 month		3 months		6 months		*p* value		
	Mean	SD	Mean	SD	Mean	SD	Mean	SD	1 m vs. BL	3 m vs. BL	6 m vs. BL
MMSE	22.10	4.35	24.16	4.87	24.31	5.24	24.70	5.25	<0.001*	<0.001*	<0.001*
ADAS-Cog	21.99	10.90	18.13	11.04	17.16	11.37	16.54	10.76	<0.001*	<0.001*	<0.001*
IADL	16.08	3.58	15.69	3.09	15.65	3.21	15.50	2.89	0.098	0.041*	0.223
GDS	5.58	5.49	3.86	3.91	3.33	3.60	3.43	4.01	0.002*	<0.001*	0.007*
NPI	1.82	8.32	0.82	2.73	1.17	3.24	0.52	1.56	0.291	0.441	0.328
QOL-AD	33.14	6.85	33.63	5.48	34.60	5.82	33.91	6.29	0.413	0.022*	0.226

SD: standard deviation; BL: baseline; 1 m: 1 month; 3 m: 3 months; 6 m: 6 months; MMSE: minimum mental state examination; ADAS-Cog: Alzheimer’s disease assessment scale-cognition; IADL: instrumental activity of daily living; GDS: geriatric depression scale; NPI: neuropsychiatric inventory; QOL-AD: quality of life in Alzheimer's disease. *p < 0.05.

### Changes of Local Spontaneous Brain Activity Following Pharmacologic Treatment

**FIGURE 2:**
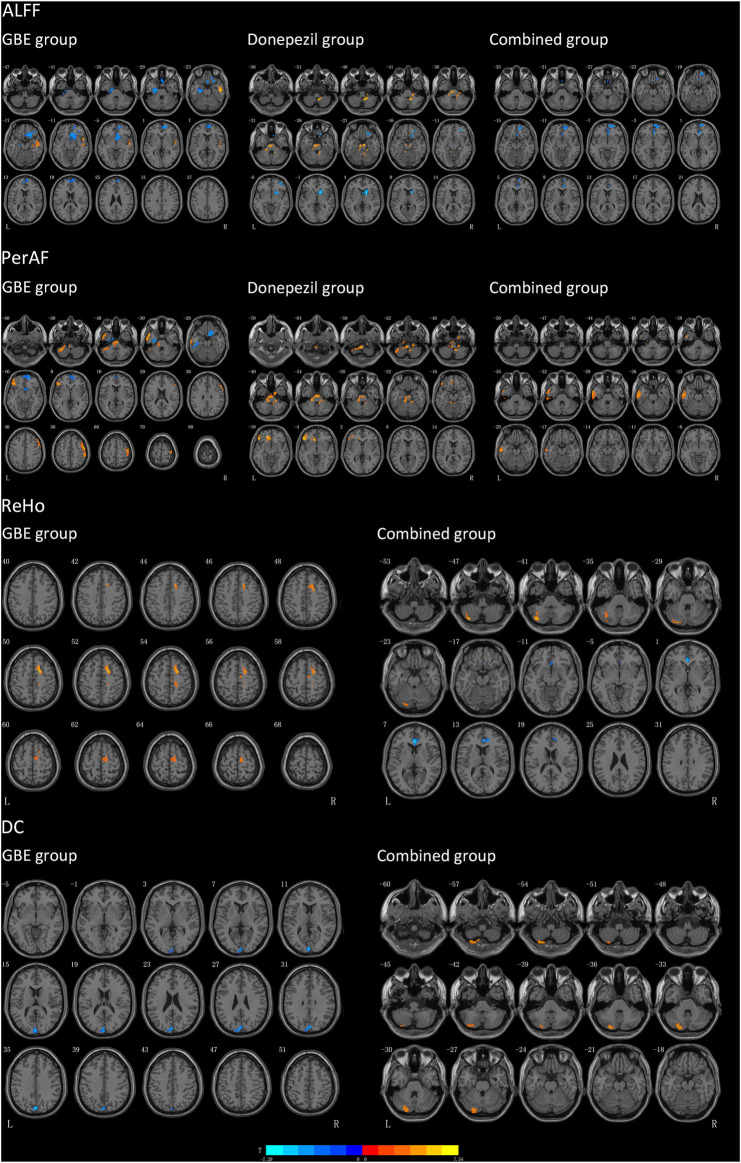
Results of brain regions demonstrate significant differences in three groups pre-and post-intervention. The pseudo-color map revealed increases in ALFF, PerAF, ReHo and DC following the intervention. Results are displayed at *p* < 0.005 corrected by GRF. T value obtained from paired *t*-test of the group. GBE: Ginkgo biloba extract; ALFF: amplitude of low-frequency fluctuation; PerAF: percent amplitude of fluctuation; DC: degree centrality; ReHo: reginal homogeneity; R: right; L: left.

As shown in Figure 2; Table 6, significant discriminative brain regions, reflected by changes of four metrics, including ALFF, PerAF, ReHo, and DC before and after treatment, were presented according to different treatment strategies.

**TABLE 6:** Brain regions with significant different values of ALFF, PerAF, ReHo, and DC of three groups before and after pharmacologic treatment.

	Clusters	MNI coordinates			Brain regions	Voxels	T values
		X	Y	Z			
ALFF	GBE group						
	Cluster 1	−24	−18	−27	Parahippocampal gyrus (L)	55	−5.03
	—				Fusiform gyrus (L)	55	−5.03
	Cluster 2	3	18	−27	Gyrus rectus (R)	69	−4.75
	Cluster 3	54	−15	−24	Middle temporal gyrus (R)	63	6.22
	Cluster 4	9	54	3	Superior frontal gyrus, medial (R)	136	−4.60
	Donepezil group						
	Cluster 1	18	−45	−45	Inferior cerebellum gyrus (R)	78	7.08
	Cluster 2	−21	−33	−33	Superior cerebellum gyrus (L)	22	5.93
	Cluster 3	9	21	−33	Superior frontal gyrus, orbital part (R)	42	-4.46
	—				Middle frontal gyrus, orbital part (R)	38	−4.46
	Cluster 4	15	9	3	Caudate nucleus (R)	77	−6.24
	—				Lenticular nucleus, putamen (R)	36	−6.24
	Combined group						
	Cluster 1	3	33	−3	Anterior cingulate and paracingulate gyri (R)	47	−5.3
	Cluster 2	9	51	−21	Superior frontal gyrus, orbital part (R)	73	−5.19
PerAF	GBE group						
	Cluster 1	−33	−42	−45	Inferior cerebellum gyrus (L)	100	5.36
	—				Superior cerebellum gyrus (L)	57	5.36
	Cluster 2	6	−15	−36	Superior cerebellum gyrus (L)	1	5.11
	Cluster 3	−51	12	−33	Inferior temporal gyrus (L)	69	5.42
	Cluster 4	−27	−12	−30	Fusiform gyrus (L)	66	−4.74
	Cluster 5	12	18	−18	Gyrus rectus (R)	45	−4.8
	Cluster 6	15	57	−12	Superior frontal gyrus, medial orbital (R)	69	−4.23
	Cluster 7	−45	30	−3	Inferior frontal gyrus, orbital part (L)	97	5.14
	Cluster 8	54	−3	51	Precental gyrus (R)	147	5.75
	—				Middle frontal gyrus (R)	101	5.75
	Donepezil group						
	Cluster 1	−12	−33	−24	Inferior cerebellum gyrus (L)	42	5.67
	Cluster 2	21	−30	−54	Inferior cerebellum gyrus (R)	50	5.45
	Cluster 3	−48	33	−3	Inferior frontal gyrus, orbital part (L)	87	7.54
	—				Inferior frontal gyrus, triangular part (L)	40	7.54
	Cluster 4	−15	39	−9	Superior frontal gyrus, medial orbital (L)	37	5.98
	Combined group						
	Cluster 1	−57	−6	−27	Inferior temporal gyrus (L)	100	4.64
	—				Middle temporal gyrus (L)	74	4.64
ReHo	GBE group						
	Cluster 1	21	3	51	Superior frontal gyrus, dorsolateral (R)	69	5.63
	—				Supplementary motor area (R)	51	5.63
	Combined group						
	Cluster 1	−39	−75	−42	Inferior cerebellum gyrus (L)	87	5.45
					Superior cerebellum gyrus (L)	43	5.45
	Cluster 2	3	36	6	Anterior cingulate and paracingulate gyri (R)	53	−7.29
	—				Anterior cingulate and paracingulate gyri (L)	28	−7.29
DC	GBE group						
	Cluster 1	0	−81	36	Cuneus (L)	104	−6.11
	Combined group						
	Cluster 1	−30	−75	−54	Inferior cerebellum gyrus (L)	54	5.33
	—				Superior cerebellum gyrus (L)	35	5.33

GBE: Ginkgo biloba extract; ALFF: amplitude of low frequency fluctuation; PerAF: percent amplitude of fluctuation; ReHo: reginal homogeneity; DC: degree centrality; MNI: Montreal Neurological Institute; L: left, R: right.

Patients in the GBE group showed a significant decrease of ALFF in the left parahippocampal gyrus, left fusiform gyrus, right gyrus rectus, and right superior frontal gyrus while an increase of ALFF in the right middle temporal gyrus. As compared to the GBE group, the discriminative brain regions in the donepezil group were quite different including the right inferior cerebellum gyrus, left superior cerebellum gyrus, right middle frontal gyrus, right caudate nucleus, and right lenticular nucleus. The combined group also showed significant changes in right anterior cingulate and paracingulate gyri. Interestingly, the three groups shared one same significantly discriminative brain regions, the right superior frontal gyrus.

In terms of PerAF, several brain regions in the GBE group showed significant discriminative including the left inferior and superior cerebellum gyrus, left inferior temporal gyrus, left fusiform gyrus, right gyrus rectus, right superior frontal gyrus, left inferior frontal gyrus, right precental gyrus, and right middle frontal gyrus. In addition, brain regions, including the bilateral inferior cerebellum gyrus, left inferior frontal gyrus, left superior frontal gyrus in the donepezil group and inferior and middle temporal gyrus in the combined group, showed a significant increase of PerAF values.

Due to the sensitivity issue, sufficiently changed signaling was only observed in the GBE group and the combined group for both ReHo and DC. A significantly increased ReHo signaling was detected in the right superior frontal gyrus and right supplementary motor area in the GBE group and the left inferior and superior cerebellum gyrus in the combined group. However, a decreased signaling was detected in the bilateral anterior cingulate and paracingulate gyri in the combined group. Furthermore, significant DC changes were presented in the left cuneus in the GBE group and left inferior and superior cerebellum gyrus in the combined group.

### Correlations Between Changes of Neuropsychological Function and Functional Magnetic Resonance Imaging Metrics

**FIGURE 3:**
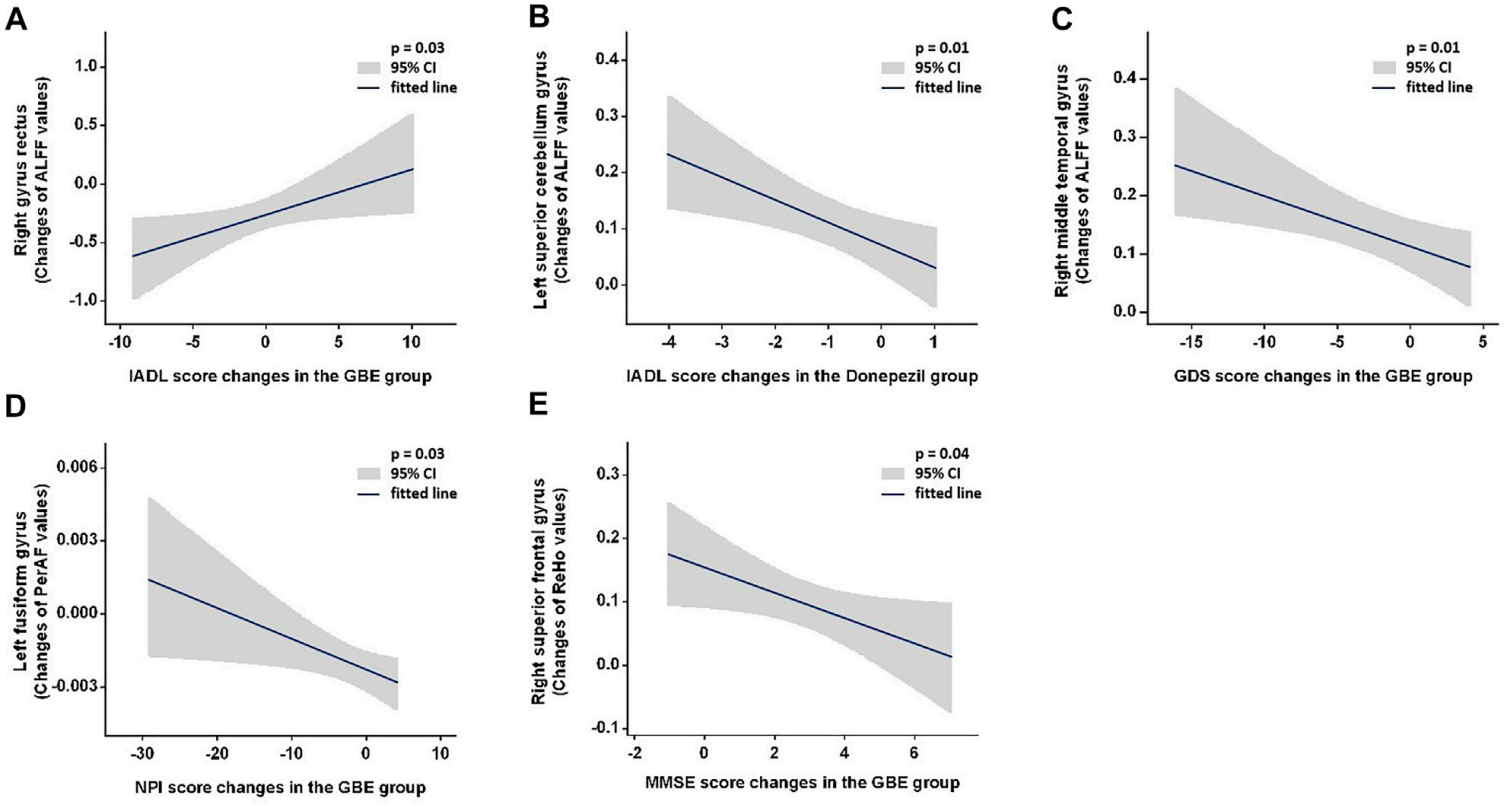
Demonstration of significant correlations between changes of neuropsychological function and fMRI metrics. GBE: Ginkgo biloba extract; ALFF: amplitude of low frequency fluctuation; PerAF: percent amplitude of fluctuation; ReHo: reginal homogeneity; IADL: instrumental activity of daily living; GDS: geriatric depression scale; NPI: neuropsychiatric inventory; MMSE: minimum mental state examination.

Figure 3 and Supplementary Tables S1–S4 show the correlations between changes of neuropsychological function and fMRI metrics. Significant positive correlations were observed between IADL changes and ALFF changes in the right gyrus rectus in the GBE group (*p* = 0.03) and negative in the left superior cerebellum gyrus in the donepezil group (*p* = 0.01). However, GDS changes was negatively correlated with ALFF changes in right middle temporal gyrus in the GBE group (*p* = 0.01). A negative correlation between NPI changes and PerAF changes in the left fusiform gyrus in the GBE group was also detected (*p* = 0.03). In addition, MMSE changes correlated negatively with ReHo changes in the right superior frontal gyrus in the GBE group (*p* = 0.04). Unfortunately, no significant correlation was found between DC changes and any neuropsychological function assessments.

## Discussion

Our results demonstrated that GBE was not superior to donepezil in terms of efficacy on AD patients’ functional recovery reflected with MMSE, ADAS-Cog, IADL, GDS, NPI, and QOL-AD across all three follow-ups. We also failed to demonstrate the superimposed efficacy when provided AD patients with both GBE and donepezil. Nonetheless, longitudinal improvement of functional impairment was observed in all three groups. Specifically, MMSE and ADAS-Cog scores increased significantly while only marginal changes were detected for IADL and QOL-AD scores. A baseline and 6-month comparison revealed a significant increase of GDS scores only in those receiving both GBE and donepezil while improvement of NPI scores were observed in patients treated with GBE or donepezil. The potential longitudinal impacts on specific adaption of the brain launched by these agents were further explored with rs-fMRI scanning and the spontaneous brain activities responded to the treatment was evaluated with four amplitude methods including ALFF, PerAF, ReHo, and DC. In addition to the adaptive changes in specific brain regions, several significant correlations should be emphasized including 1) positive correlations between IADL and ALFF changes in the right precentral gyrus right gyrus rectus in the GBE group and negative in the left superior cerebellum gyrus in the donepezil group; 2) negative correlation between GDS and ALFF changes in the right middle temporal gyrus in the GBE group; 3) negative correlation between NPI and PerAF changes in the left fusiform gyrus in the GBE group; and 4) negative correlations between MMSE and ReHo changes in the right superior frontal gyrus in the GBE group.

In the current study, we examined cognitive, behavioral, psychological, and global outcomes across three different pharmacologic strategies. As the second-generation acetylcholinesterase inhibitors, donepezil has been licensed for use in more than 90 countries after the completion of large multicenter studies (Cacabelos, 2007). Dose-dependent trials reported that a higher dose (10 mg/day) of donepezil was effective to show certain improvement of cognitive function, however benefits on a higher dose were only marginally larger than that on a lower dose (5 mg/day) in terms of ADL and clinician-related global impression (Homma et al., 2008). In addition, patients treated with a higher dose were more likely to experience adverse events or to withdraw from the trial (Birks and Harvey, 2018). For these reasons, we adopted the lower dose with which the efficacy was compared with GBE on multiple outcomes. As a preclinical drug for AD, GBE is still in the development phase and substantial efforts have been taken to verify its efficacy. Unfortunately, two randomized, double-blind, placebo-controlled trials failed to show adequate efficacy (Schneider et al., 2005; McCarney et al., 2008). The Cochrane review summarized that the evidence was not sufficient to support its benefits on cognitive impairment (Birks and Grimley Evans, 2009). Nonetheless, the current study showed non-inferiority results of GBE vs. donepezil across all comparisons at each follow-up. In addition to QoL, the longitudinal analysis also showed promising results supporting the efficacy of GBE. Taken together, GBE might be anticipated to present superimposed efficacy with the use of donepezil. However, the combined group showed no significant improvement according to the results of multiple comparisons. Therefore, the combined application of GBE and donepezil seems to be unnecessary. Our attention was then shifted to the observed discrepancies of multiple outcomes between GBE vs. donepezil. The upcoming challenge becomes the clinical selection of these two drugs with which different brain regions may be impacted and then spread into improvement of specific functional recovery.

The gyrus rectus is located at the medial margin of the inferior surface of the frontal lobe and is associated with memory and behavioral function (Joo et al., 2016; Destrieux et al., 2017). Impaired gyrus rectus function with decreased spontaneous brain activities was previously demonstrated in patients with AD as compared to the healthy controls, which might reflect a common pathological condition in patients with AD (Sheline et al., 2010; Cheng et al., 2019). However, its linkage to the neurotoxicity of the amyloid *β* protein proposed a possible treatment hallmark that this condition might be reversed through GBE treatment (Sheline et al., 2010). Our results were consistent with these findings and hypotheses. Specifically, we observed decreased ALFF values in the right gyrus rectus which was positively correlated with improved IADL scores after GBE treatment, indicating that benefits in IADL may be partially attributed to the GBE-induced compromise of ALFF reduction.

According to the literature review, few studies investigated the adaptive changes in the middle temporal gyrus after AD. Abnormal ALFF values in AD patients, either increased or decreased, were observed in a recent study (Liu et al., 2014). The authors hypothesized that abnormalities may be associated with specific frequency bands in ALFF measurements. Therefore, they divided the low frequency range into several distinct bands and found decreased ALFF value in the slow-5 band (0.01–0.027 Hz) and increased ALFF value in the slow-4 band (0.027–0.073 Hz). They concluded that a specific frequency band would contribute to sensitive detection of spontaneous brain activity abnormalities. Although the current study observed increased ALFF values in the right middle temporal gyrus after GBE treatment, it is unlikely to draw a conclusion because our ALFF measurement was performed in a standardized way (frequency band of 0.01–0.08 Hz). Although a significant correlation was detected, the improvement of GDS score was difficult to be explained by the change of right middle temporal gyrus function since this specific brain region was previously reported to be involved in verbal or semantic cognition and associated with oral short-term memory (Vandenberghe et al., 1996). Both change of spontaneous brain activity in the middle temporal gyrus and its interaction with depression status following pharmacologic treatment need to be further clarified with well-designed clinical studies.

As a newly developed metric, PerAF is an analog to the percent signal change and a straightforward measurement of BOLD signal fluctuations during the resting state (Jia et al., 2020). It has been proven to be more reliable and sensitive than ALFF and fractional ALFF (fALFF) in a test-retest reliability analysis. Nonetheless, it has not been widely used in fMRI studies. Therefore, as compared to the healthy controls increased values in the fusiform gyrus of AD patients were only reported in the ALFF and ReHo measurement (Dai et al., 2012). We presented decreased PerAF values indicating that GBE was effective in compromising left fusiform gyrus function to some extent. Additionally, the fusiform gyrus was reported to be linked to various neurological phenomena including synesthesia, dyslexia, and prosopagnosia. Along with the results of correlation analysis, it is reasonable to document a positive interaction between reversed PerAF values in the left fusiform gyrus and improved neuropsychiatric status following GBE treatment.

We only observed one significant correlation in the donepezil group. As documented in previous studies, the cerebellum is involved in motor and balance as well as cognitive functions. The spontaneous brain activities in this region were reported to present decreased trends in patients with AD (Gottwald et al., 2003; Yang et al., 2018). Although the current study showed an increased trend of ALFF values in the left superior cerebellum gyrus after the treatment of donepezil, its negative correlation with improved IADL ability suggested that the functional improvement might not be directly subject to the recovery of cerebellum function while a potential effect inferred by the altered functional connection of salience network to the whole brain induced by donepezil (Cai et al., 2020). Similar situations can be casted to the observed negative correlations between increased ReHo values in the right superior frontal gyrus and improved MMSE scores. Further studies are warranted to clarify how this drug improves the IADL ability.

This study is not without limitations. Subsequent efforts had been donated during the enrollment period, for example, to minimize dropouts dosage selection of donepezil had been carefully considered according to both literature evidence and clinical experience. However, the dropout rate in the donepezil group was relatively high. Such attrition could have biased the results of multiple comparisons. In addition, the variation of cognitive impairment severity may lead to confounding bias although the average baseline MMSE values were comparable and the SDs were small across the three groups. Nonetheless, this limitation may be balanced with the application of multilevel imaging metrics including ALFF, PerAF, ReHo, and DC. Their discriminative sensitive features allowed the capture of potential significant changes of BOLD signaling in specific brain regions. Indeed, without healthy controls the compromised reduction or increase of fMRI metrics cannot be clearly defined and the impact of natural history of AD cannot be totally ruled out. Finally, due to the heterogeneity of pharmacological (e.g., type, dosage and duration) and analytic strategies, a generalization of the results is challenging.

## Conclusion

In conclusion, based on the results of inter-and intragroup comparison, GBE was comparable with donepezil in the improvement of cognitive, behavioral, psychological, and global functions in patients with AD while the combined application of GBE and donepezil seems unnecessary. Nonetheless, the acting mechanisms of these two drugs were discriminative. Although the IADL improvement might not be directly revealed with the recovery of cerebellum function following donepezil treatment, GBE-mediated improvement of functional recovery was potentially linked to the decreased ALFF values in the right gyrus rectus and decreased PerAF values in the left fusiform gyrus. These featured variations of imaging metrics in specific brain regions may serve as potential biomarkers in the monitoring of the therapeutic efficacy of GBE. Well-designed studies are warranted to fully investigate the efficacy and mechanisms of pharmacologic treatment on functional recovery in patients with AD.

## Data Availability

The original contributions presented in the study are included in the article Supplementary Material; further inquiries can be directed to the corresponding author.
