# Supplementary material for: Ginkgo Biloba Extract Is Comparable With Donepezil in Improving Functional Recovery in Alzheimer’s Disease: Results From a Multilevel Characterized Study Based on Clinical Features and Resting-State Functional Magnetic Resonance Imaging
Source: Front Pharmacol. 2021 Aug 3;12:721216. doi: 10.3389/fphar.2021.721216 (PMC8369572; doi:10.3389/fphar.2021.721216)
Supplement: Supplementary file 1 [file DataSheet1.DOCX]

**Supplemental materials**

**Table S1 Correlations between ALFF value changes and neuropsychological test score changes**

| **Clusters** |  | **MMSE** | **ADAS-Cog** | **IADL** | **GDS** | **NPI** | **QOL-AD** |
| --- | --- | --- | --- | --- | --- | --- | --- |
| **GBE group** |  |  |  |  |  |  |  |
| Cluster 1 (-24, -18, -27) | *r* | -0.39 | 0.39 | 0.39 | 0.38 | 0.02 | -0.19 |
|  | *p* | 0.09 | 0.09 | 0.09 | 0.10 | 0.92 | 0.43 |
| Cluster 2 (3, 18, -27) | *r* | -0.35 | 0.38 | 0.48 | 0.14 | -0.07 | 0.17 |
|  | *p* | 0.13 | 0.10 | 0.03* | 0.55 | 0.78 | 0.49 |
| Cluster 3 (54, -15, -24) | *r* | 0.19 | -0.26 | -0.32 | -0.55 | 0.00 | 0.08 |
|  | *p* | 0.43 | 0.26 | 0.17 | 0.01* | 0.99 | 0.74 |
| Cluster 4 (9, 54, 3) | *r* | -0.27 | 0.16 | 0.26 | 0.35 | -0.03 | 0.06 |
|  | *p* | 0.25 | 0.49 | 0.27 | 0.13 | 0.91 | 0.81 |
| **Donepezil group** |  |  |  |  |  |  |  |
| Cluster 1 (18, -45, -45) | *r* | -0.05 | -0.20 | -0.41 | 0.07 | 0.05 | -0.21 |
|  | *p* | 0.86 | 0.44 | 0.10 | 0.78 | 0.86 | 0.42 |
| Cluster 2 (-21, -33, -33) | *r* | -0.02 | -0.15 | -0.60 | -0.02 | -0.12 | -0.01 |
|  | *p* | 0.93 | 0.56 | 0.01* | 0.95 | 0.64 | 0.97 |
| Cluster 3 (9, 21, -33) | *r* | -0.15 | 0.40 | -0.07 | -0.08 | -0.20 | 0.21 |
|  | *p* | 0.56 | 0.11 | 0.80 | 0.75 | 0.45 | 0.42 |
| Cluster 4 (15, 9, 3) | *r* | -0.37 | -0.20 | -0.36 | 0.15 | -0.04 | 0.07 |
|  | *p* | 0.14 | 0.45 | 0.16 | 0.56 | 0.89 | 0.78 |
| **Combined group** |  |  |  |  |  |  |  |
| Cluster 1 (3, 33, -3) | *r* | -0.07 | 0.09 | 0.24 | 0.02 | -0.03 | 0.18 |
|  | *p* | 0.77 | 0.71 | 0.30 | 0.92 | 0.90 | 0.44 |
| Cluster 2 (9, 51, -21) | *r* | 0.28 | 0.12 | 0.31 | -0.04 | -0.04 | 0.19 |
|  | *p* | 0.24 | 0.60 | 0.18 | 0.85 | 0.88 | 0.42 |

*ALFF: amplitude of low frequency fluctuation; MMSE: minimum mental state examination; ADAS-Cog: Alzheimer's disease assessment scale-cognition; IADL: instrumental activity of daily living; GDS: geriatric depression scale; NPI: neuropsychiatric inventory; QOL-AD: quality of life in Alzheimer's disease; GBE: ginkgo biloba extract. *p<0.05.*

**Table S2 Correlations between PerAF value changes and neuropsychological test score changes**

| **Clusters** |  | **MMSE** | **ADAS-Cog** | **IADL** | **GDS** | **NPI** | **QOL-AD** |
| --- | --- | --- | --- | --- | --- | --- | --- |
| **GBE group** |  |  |  |  |  |  |  |
| Cluster 1 (-33, -42, -45) | *r* | 0.34 | -0.16 | -0.34 | -0.17 | 0.01 | -0.12 |
|  | *p* | 0.15 | 0.50 | 0.14 | 0.49 | 0.95 | 0.62 |
| Cluster 2 (6, -15, -36) | *r* | 0.30 | -0.26 | -0.37 | -0.16 | -0.09 | -0.21 |
|  | *p* | 0.20 | 0.27 | 0.11 | 0.49 | 0.71 | 0.37 |
| Cluster 3 (-51, 12, -33) | *r* | 0.01 | -0.14 | -0.32 | -0.23 | 0.00 | -0.09 |
|  | *p* | 0.96 | 0.57 | 0.17 | 0.33 | 0.99 | 0.70 |
| Cluster 4 (-27, -12, -30) | *r* | 0.08 | -0.18 | -0.07 | -0.04 | -0.49 | 0.09 |
|  | *p* | 0.74 | 0.44 | 0.76 | 0.88 | 0.03* | 0.71 |
| Cluster 5 (12, 18, -18) | *r* | -0.24 | 0.10 | 0.24 | 0.37 | -0.37 | -0.17 |
|  | *p* | 0.31 | 0.66 | 0.32 | 0.11 | 0.11 | 0.47 |
| Cluster 6 (15, 57, -12) | *r* | -0.25 | 0.11 | 0.15 | 0.19 | -0.30 | 0.05 |
|  | *p* | 0.29 | 0.66 | 0.54 | 0.42 | 0.19 | 0.84 |
| Cluster 7 (-45, 30, -3) | *r* | 0.38 | -0.32 | -0.39 | -0.17 | -0.30 | -0.08 |
|  | *p* | 0.10 | 0.17 | 0.09 | 0.48 | 0.21 | 0.74 |
| Cluster 8 (54, -3, 51) | *r* | 0.26 | -0.07 | -0.28 | -0.23 | 0.26 | 0.14 |
|  | *p* | 0.27 | 0.77 | 0.23 | 0.34 | 0.27 | 0.57 |
| **Donepezil group** |  |  |  |  |  |  |  |
| Cluster 1 (-12, -33, -24) | *r* | -0.08 | -0.18 | 0.20 | -0.35 | -0.08 | 0.05 |
|  | *p* | 0.77 | 0.50 | 0.43 | 0.17 | 0.77 | 0.85 |
| Cluster 2 (21, -30, -54) | *r* | 0.36 | -0.41 | -0.17 | 0.01 | 0.26 | -0.43 |
|  | *p* | 0.15 | 0.10 | 0.51 | 0.97 | 0.32 | 0.08 |
| Cluster 3 (-48, 33, -3) | *r* | 0.28 | 0.04 | 0.25 | 0.23 | 0.24 | -0.06 |
|  | *p* | 0.27 | 0.89 | 0.33 | 0.37 | 0.35 | 0.81 |
| Cluster 4 (-15, 39, -9) | *r* | 0.21 | -0.42 | -0.05 | 0.32 | 0.17 | -0.34 |
|  | *p* | 0.42 | 0.09 | 0.85 | 0.21 | 0.51 | 0.18 |
| **Combined group** |  |  |  |  |  |  |  |
| Cluster 1 (-57, -6, -27) | *r* | -0.08 | 0.29 | 0.07 | 0.36 | 0.18 | 0.21 |
|  | *p* | 0.73 | 0.22 | 0.78 | 0.12 | 0.44 | 0.38 |

*PerAF: percent amplitude of fluctuation; MMSE: minimum mental state examination; ADAS-Cog: Alzheimer's disease assessment scale-cognition; IADL: instrumental activity of daily living; GDS: geriatric depression scale; NPI: neuropsychiatric inventory; QOL-AD: quality of life in Alzheimer's disease; GBE: ginkgo biloba extract. *p<0.05.*

**Table S3 Correlations between ReHo value changes and neuropsychological test score changes**

| **Clusters** |  | **MMSE** | **ADAS-Cog** | **IADL** | **GDS** | **NPI** | **QOL-AD** |
| --- | --- | --- | --- | --- | --- | --- | --- |
| **GBE group** |  |  |  |  |  |  |  |
| Cluster 1 (21, 3, 51) | *r* | -0.47 | 0.41 | 0.27 | 0.31 | 0.05 | -0.33 |
|  | *p* | 0.04* | 0.07 | 0.24 | 0.18 | 0.83 | 0.16 |
| **Combined group** |  |  |  |  |  |  |  |
| Cluster 1 (-39, -75, -42) | *r* | -0.16 | -0.06 | 0.34 | 0.17 | 0.11 | 0.09 |
|  | *p* | 0.49 | 0.81 | 0.14 | 0.48 | 0.65 | 0.71 |
| Cluster 2 (3, 36, 6) | *r* | 0.26 | 0.03 | -0.11 | 0.00 | 0.07 | 0.39 |
|  | *p* | 0.27 | 0.90 | 0.65 | 0.99 | 0.76 | 0.09 |

*ReHo: reginal homogeneity; MMSE: minimum mental state examination; ADAS-Cog: Alzheimer's disease assessment scale-cognition; IADL: instrumental activity of daily living; GDS: geriatric depression scale; NPI: neuropsychiatric inventory; QOL-AD: quality of life in Alzheimer's disease; GBE: ginkgo biloba extract. *p<0.05.*

**Table S4 Correlations between DC value changes and neuropsychological test score changes**

| **Clusters** |  | **MMSE** | **ADAS-Cog** | **IADL** | **GDS** | **NPI** | **QOL-AD** |
| --- | --- | --- | --- | --- | --- | --- | --- |
| **GBE group** |  |  |  |  |  |  |  |
| Cluster 1 (0, -81, 36) | *r* | -0.29 | 0.11 | 0.02 | 0.31 | 0.44 | 0.04 |
|  | *p* | 0.21 | 0.63 | 0.93 | 0.18 | 0.05 | 0.86 |
| **Combined group** |  |  |  |  |  |  |  |
| Cluster 1 (-30, -75, -54) | *r* | 0.28 | -0.22 | -0.34 | -0.44 | -0.32 | 0.35 |
|  | *p* | 0.22 | 0.36 | 0.15 | 0.05 | 0.18 | 0.13 |

*DC: degree centrality; MMSE: minimum mental state examination; ADAS-Cog: Alzheimer's disease assessment scale-cognition; IADL: instrumental activity of daily living; GDS: geriatric depression scale; NPI: neuropsychiatric inventory; QOL-AD: quality of life in Alzheimer's disease; GBE: ginkgo biloba extract.*
